# Do third age adults benefit equally in well-being from activity participation? The moderating effect of financial status

**DOI:** 10.1371/journal.pone.0330898

**Published:** 2025-09-03

**Authors:** Nan Qin, Daniel W. L. Lai

**Affiliations:** 1 Department of Applied Sociology, Guangdong University of Finance and Economics, Guangzhou, China; 2 Department of Social Work, Hong Kong Baptist University, Kowloon, Hong Kong; Victoria University, AUSTRALIA

## Abstract

The relationship between activity participation and well-being has been well documented for third age adults. However, little has been known about how the financial status influences this relationship. This study aims to investigate the moderating effect of financial status on the association of activity level with subjective happiness and quality of life among third age adults. Systematic sampling was used to select a sample of 304 adults aged 50 and older from an active ageing institute in Hong Kong. Hierarchical regression analyses indicated that activity level was a salient predictor for subjective happiness and quality of life when controlling for socio-demographics. It was not salient anymore when its interaction term with financial status was added to the model. The interaction term significantly predicted quality of life but not subjective happiness. When the data were separated by financial status, activity level saliently predicted subjective happiness and quality of life for participants with good or very good financial statuses but not for those with poor or average statuses. The results suggested that financial status played a moderating role in the relationship between activity participation and well-being. Specifically, third age adults with limited financial resources need more welfare support to benefit from activity participation.

## Introduction

Well-being of older people is the most common focal point in contemporary gerontological research. Subjective happiness and quality of life have been commonly reported as well-being indicators related to activity participation [1,2]. Previous research provided empirical evidence on the positive association between subjective happiness and activity participation of older people, including physical, leisure, social and solitary activities [3,4] and the negative relationship between sedentary behavior and happiness [5]. The documented activity domains increasing or maintaining the quality of life among older adults included physical exercise, Instrumental Activity of Daily Living (IADL), leisure activity (e.g., gardening, hobbies, reading books), and social activity [6].

As the Activity Theory of Ageing asserted [7], activity participation is a prerequisite for older people to achieve greater well-being. However, this tenet has been challenged for a lack of attention to personal backgrounds. Previous research suggested the necessity of refining the Activity Theory of Ageing by considering possible moderators of the relationship between activity participation and well-being of older people, including age, gender, life situation, and so forth [8]. As a remedy, the Social Production Function Theory [9–11] considers individual differences (including financial status) to disentangle the complex relationship between activity participation and well-being. This theory assumes that people produce their well-being (physical and social well-being) as much as possible through achieving important instrumental goals (stimulation, comfort, status, behavioural confirmation, and affection) within the limitations of resources and constraints. People’s activities are means to produce instrumental goals and further their well-being. However, engaging in activities is subject to the resources people possess, including finances. For example, income determines the access people have to activities for stimulation and comfort. Meanwhile, financial status is an essential component of one’s overall condition, so it may well influence well-being [12–14]. Existing empirical research has shed light on the relations of financial status to activity and to well-being (represented by subjective happiness and quality of life).

### The influence of financial status on activity participation

Financial status determines people’s opportunities for activities (e.g., the amount and types of activities). Financial expense is requisite for older people to participate in activities, such as tourism, gardening, painting, education and so forth. Poverty would reduce access to costly activities [15]. Empirical research found that income predicted higher levels of physical activity [16] and out-of-home social and cultural activities [17] in older adults. Generally, it is supposed that when lacking financial resources, older people have to commit themselves to effortful activities (e.g., work, housework, caregiving, etc.) or live a withdrawn lifestyle.

### The influence of financial status on subjective happiness and quality of life

Financial resources are deemed as one of the most robust predictors of subjective well-being in older adults [18]. However, evidence on the relationship between financial status and happiness is mixed. A study based on a large-scale survey indicated that family income and other wealth had significant and positive effects on happiness of older Chinese people [19]. However, another study [20] revealed that subjective happiness would not always grow with the improvement of financial status. It verified the “Easterlin Paradox” [21], i.e., a phenomenon that happiness would stop growing at a certain level of richness. Nevertheless, it is undeniable that financial resources (e.g., income) are essential for maximizing one’s subjective happiness [22] and quality of life” [18]. Income was positively correlated with the quality of life in older adults [23] while the financial stresses older people face might harm their quality of life [24].

### The moderators of the relations of activity participation to subjective happiness and to quality of life

Do older people benefit equally in well-being from activity participation? The answer might be “no.” A critical literature review summarized the potential moderators in the association between subjective well-being and participation in activities in later life [8] but little has been known on the moderating effect of financial status on the relationship between activity participation and subjective happiness and quality of life. Previous research on the general population showed that income might moderate the relationship between arts and cultural activities and happiness [25], the relationship between volunteering and happiness [26], and the relationship between active living behaviors and the health-related quality of life [27]. More studies tended to ignore that the financial status of older adults might create additional complications for the relationship, not only in the area of affordability of activity participation, but also in the direct well-being outcomes of activity participation.

### The present study

This study aims to determine whether financial status would moderate the impacts of activity levels on subjective happiness and quality of life of third age adults (people in the period of retirement but before dependence). According to theoretical viewpoints and empirical studies aforementioned, it is *hypothesized that third age adults with a good financial status are more likely to report subjective happiness and good quality of life than those with an average or poor financial status as the result of activity participation.*

## Methods

### Research design and data collection

This study employed a quantitative approach, with data collected from adults aged 50 and older in Hong Kong via telephone survey. The survey sample was recruited from registrants in non-interventional programmes (including interest classes, talks, etc.) run by an institute of active ageing affiliated with a university in Hong Kong from Dec. 1^st^, 2016 to Mar. 31^st^, 2017. A total of 1,729 registrants were ranked based on their participation hours (ranging from one to 263 hours with a mean of 15.63 and a standard deviation of 30.64) in the institute’s programmes. A systematic sampling method was used to select every other registrant on this list as potential survey participants. A total of 869 cases were selected for the survey. A maximum of three calls were made to each contact number by trained interviewers from a professional survey team in 2017, resulting in the identification of 604 valid numbers. Of these, 304 third age adults gave oral consent to participate in the survey and completed the interview, resulting in a response rate of 50.3 percent (successful cases/ valid phone numbers). Each interview lasted for approximately 30 minutes. The authors checked the data and recordings to ensure data validity. A comparison of basic age, gender, and educational backgrounds of the successful and refused cases showed no significant difference in their demographic characteristics.

### Instruments

Participants provided information on their socio-demographic characteristics, perceived health, level of activity participation, subjective happiness, and quality of life.

*Socio-demographics and health.* Seven demographic variables were included. See details in Table 1. Participants rated their physical and mental health on a five-point scale ranging from ‘very poor’ (one) to ‘very good’ (five).

**Table 1:** Participants’ socio-demographics and activity level by financial status (N = 304).

	Total (304, 100%)		Poor or average financial status (189, 62.2%)		Good or very good financial status (115, 37.8%)		Financial difference	
	n	%	n	%	n	%	χ2	p
**Gender**								
Male	92	30.3	54	58.7	38	41.3	0.68	.410
Female	212	69.7	135	63.7	77	36.3		
**Education**								
Primary or below	21	6.9	17	81.0	4	19.0	11.86	.003
Secondary	127	41.8	89	70.1	38	29.9		
College or above	156	51.3	83	53.2	73	46.8		
**Have children**								
No	65	21.4	46	70.8	19	29.2	2.60	.107
Yes	239	78.6	143	59.8	96	40.2		
**Living alone**								
No	259	85.2	157	60.6	102	39.4	1.80	.180
Yes	45	14.8	32	71.1	13	28.9		
**Retirement**								
No	76	25.3	44	57.1	33	42.9	1.11	.292
Yes	227	74.7	145	63.9	82	36.1		
**Physical health**								
Poor	14	4.6	12	85.7	2	14.3	38.95	<.001
Average	140	46.0	110	78.6	30	21.4		
Good	92	30.3	42	45.7	50	54.3		
Very good	58	19.1	25	43.1	33	56.9		
**Mental health**								
Poor	6	2.0	5	83.3	1	16.7	30.55	<.001
Average	102	33.5	83	82.2	18	17.8		
Good	112	36.8	63	55.8	50	44.2		
Very good	84	27.6	38	45.2	46	54.8		
**Activity level**								
Low	81	26.6	50	61.7	31	38.3	2.49	.288
Moderate	142	46.7	94	66.2	48	33.8		
High	81	26.6	45	55.6	36	44.4		

Bivariate correlation analyses revealed that activity level was significantly and positively related to subjective happiness (r = .232, p < .001), and quality of life (r = .267, p < .001). Financial status was also significantly associated with greater subjective happiness (r = .209, p < .001), and higher quality of life (r = .263, p < .001).

*Level of activity participation.* Participants were asked, “What was your activity level in participating in activities (things keeping them active physically, mentally, spiritually or socially) in the previous year?” Responses were coded as ‘low’ (one), ‘moderate’ (two), and ‘high’ (three).

*Subjective happiness.* The four-item subjective happiness scale [28] was used to assess participants’ perception of global happiness on a seven-point scale ranging from ‘not at all’ (one) to ‘a great deal’ (seven). The items and responses are “1. I consider myself a very happy person; 2. Compared to most of my peers, I consider myself happier; 3. Some people are generally very happy. They enjoy life regardless of what is going on, getting the most out of everything. To what extent does this describe you? and 4. Some people are generally not very happy. Although they are not depressed, they never seem as happy as they might be. To what extent does this describe you?” This scale demonstrated good internal consistency (0.79 to 0.94) in previous research, including studies on older adults [28]. The Chinese version has been translated and validated by Nan and his team [29], resulting in satisfactory reliability and validity. The total scale score was generated by averaging all item scores, with higher scores representing greater subjective happiness. The internal reliability of this scale in the present study was acceptable (α = 0.72).

*Quality of life*. Participants’ quality of life was measured by the 24-item World Health Organization Quality of Life Instrument-Older Adults Module (WHOQOL-OLD). This scale consists of six facets, including sensory abilities (i.e., rating sensory functioning), autonomy (i.e., freedom to make their own decisions), past, present, and future activities (i.e., happy with things to look forward to), social participation (i.e., satisfied with the opportunity to participate in the community), death and dying (i.e., scared of dying), and intimacy (i.e., the opportunity to love) [30]. Previous studies have verified this as a reliable instrument (alpha 0.72–0.88). The Chinese version used in this study has been validated [31]. The score for each item was aggregated to form the scale score, with higher scores indicating better quality of life. A satisfactory internal reliability coefficient of 0.86 was reported for this measure.

### Ethical approval

The Human Research Ethics Committee for Non-Clinical Faculties at the Hong Kong Polytechnic University where the authors were affiliated, issued ethical approval for this study (Reference Number: HSEARS20161017004). The Committee knew that participants were registrants of active ageing programmes who had normal cognitive capacity and approved the following consent procedure. Participants responded to the telephone interview and determined whether to participate in this research personally after knowing the detailed information about the research. All telephone interviews (including participants’ verbal consents) were recorded and checked by the authors. Best practices and ethical standards have been met throughout the research design and execution.

### Data analysis

SPSS 20.0 was used for the analyses. After conducting basic descriptive analyses (including bivariate correlations, T-tests and Chi-square tests), multivariable regressions were used to examine the prediction of activity levels, financial status and their interaction term (computed by multiplying activity levels and financial status to test the moderating effect) on subjective happiness and quality of life.

## Results

The final sample had an average age of 63.46 years old (SD = 6.88). Participants with a good or very good financial status were younger than those with a poor or average financial status (t = 3.77, p < .001). Table 1 depicts participants’ other profiles according to financial status. According to t-tests and Chi-square tests, participants with a good or very good financial status were more likely to attain higher education (χ^2^ = 11.86, p = .003), and perceived better physical health (χ^2^ = 38.95, p < .001) and mental health (χ^2^ = 30.55, p < .001), when compared to those with a poor or average financial status. There were no significant differences in activity levels according to financial status (p > .05).

Hierarchical regression models were tested to examine the contribution of activity level to subjective happiness and quality of life as shown in Table 2. The major variables were entered into the first block, and the interaction term of financial status and activity level was entered into the second block. These models explained 19.0% of the variance in subjective happiness and 40.0% of the variance in quality of life. Activity level was a significant positive predictor of subjective happiness (β = .159, p = .004) and quality of life (β = .151, p = .002) in the first block but financial status was not. Better mental health (β = .252, p = .002) was a salient predictor of greater subjective happiness, while a younger age (β = −.160, p = .005), higher education (β = .109, p = .031), and better mental health (β = .402, p < .001) significantly predicted a better quality of life. In the second block, the interaction term significantly predicted quality of life (β = .516, p = .008), but not subjective happiness (p > .05).

**Table 2:** Hierarchical regression on subjective happiness and quality of life.

	Predicting subjective happiness		Predicting quality of life	
	Block 1 socio-demographics	Block 2 interaction effect	Block 1 socio-demographics	Block 2 interaction effect
Predictor	Standardized coefficients beta		Standardized coefficients beta	
Age	.039	.045	−.160**	−.151**
Gender	.058	.059	−.077	−.076
Education	.045	.043	.109*	.106*
Having children	−.008	−.003	.002	.010
Living alone	−.095	−.100	−.087	−.094
Retirement	−.036	−.047	.029	.012
Financial status	.097	−.136	.042	−.296*
Physical health	.065	.074	.088	.101
Mental health	.252**	.246**	.402***	.393***
Activity level	.159**	−.087	.151**	−.206
Financial status * activity level		.355		.516**
*R* ^*2*^	.183	.190	.385	.400
*F*	6.584***	6.247***	18.337***	17.681***
*∆R* ^*2*^		.007		.015
*∆F*		2.527		7.224**

Note. Listwise *N* = 304. * *p* < .05. ** *p* < .01. *** *p* < .001. In the above models, Block 1 includes the following variables: age, gender, education, presence of children, living alone, retirement status, financial status, physical health, mental health, and activity level; Block 2 adds the interaction term of financial status and activity level in addition to the variables in Block 1.

**Fig 1:**
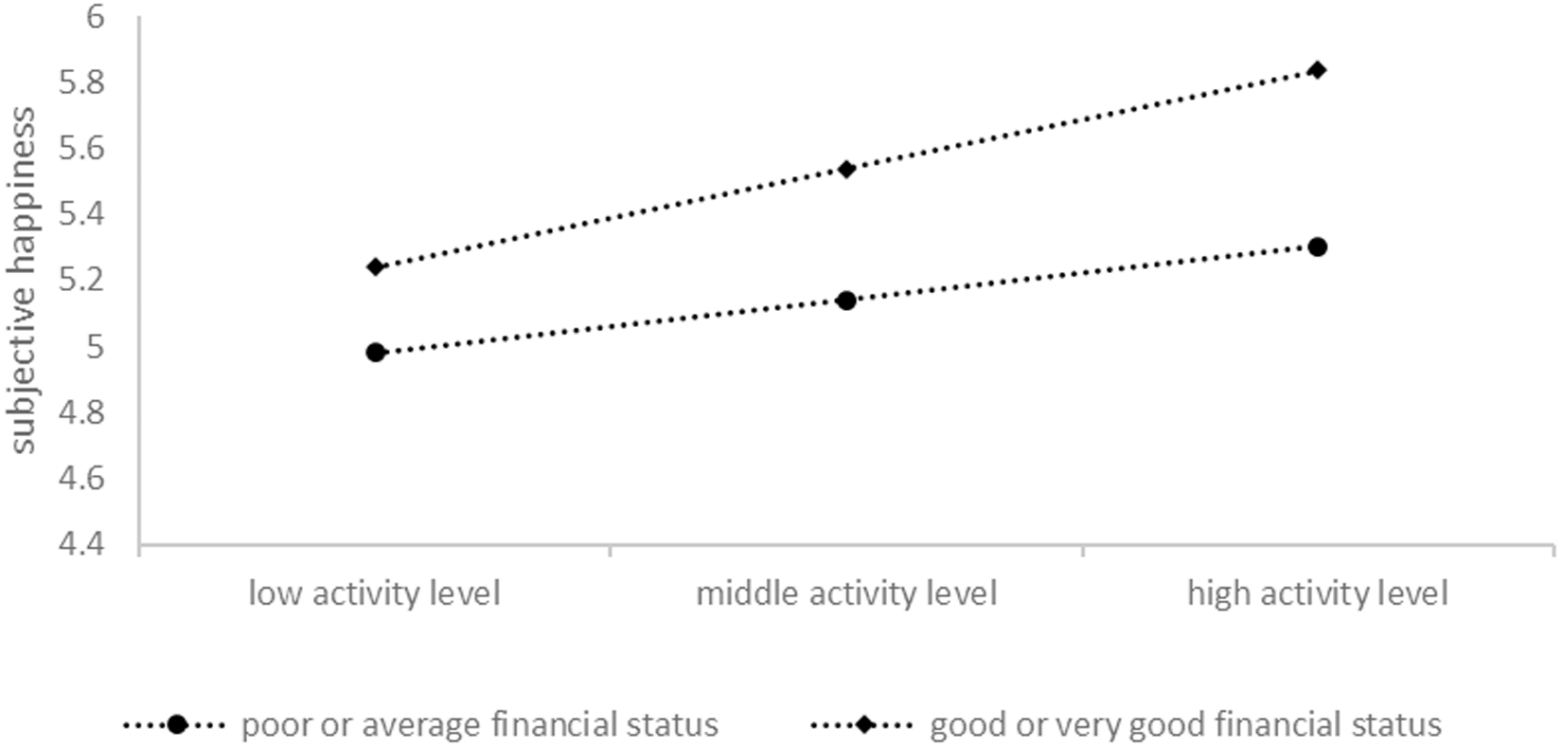
Visual plot of the interaction of activity level and financial status predicting subjective happiness.

**Fig 2:**
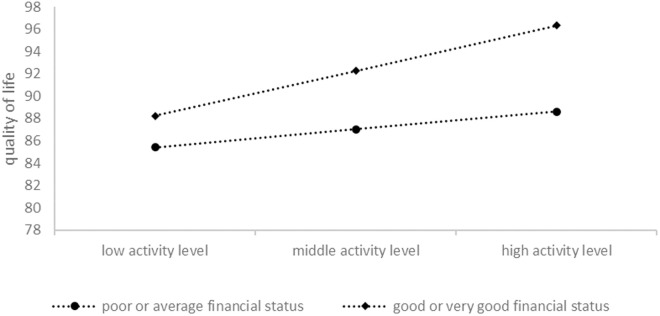
Visual plot of the interaction of activity level and financial status predicting quality of life.

To examine the specific effect of financial status on the association between activity level and subjective happiness, and the association between the activity level and quality of life, separate multivariable regressions were conducted for participants with different financial statuses, as shown in Table 3 and Table 4. Among participants with good or very good financial status, activity level was a significant positive predictor of both subjective happiness (β = .344, p < .001) and quality of life (β = .337, p < .001). In contrast, for participants with poor or average financial status, activity level was no longer significant (p > .05). However, according to the slope analyses in Fig 1 and Fig 2, the moderation effects of financial status exist in the relationships between activity level and quality of life but not in the relationships between activity level and subjective happiness.

**Table 3:** Multivariable regression on subjective happiness and quality of life for participants with good or very good financial status.

	Predicting subjective happiness	Predicting quality of life
Predictor	Standardized coefficients beta	
Age	.122	−.080
Gender	.066	.025
Education	−.032	.155
Having children	−.032	.133
Living alone	−.083	.006
Retirement	−.013	−.014
Physical health	−.184	.034
Mental health	.300*	.294**
Activity level	.344***	.337***
*R* ^*2*^	.200	.326
*F*	2.922**	5.630***

Note. Listwise *N*=115. * *p* <.05. ** *p* <.01. *** *p* <.001.

**Table 4:** Multivariable regression on subjective happiness and quality of life for participants with poor or average financial status.

	Predicting subjective happiness	Predicting quality of life
Predictor	Standardized coefficients beta	
Age	.018	−.185**
Gender	.043	−.147*
Education	.081	.079
Having children	.004	−.043
Living alone	−.104	−.115
Retirement	−.070	.025
Physical health	.248*	.154
Mental health	.157	.438***
Activity level	.044	.029
*R* ^*2*^	.185	.405
*F*	4.508***	13.564***

Note. Listwise *N*=189. * *p* <.05. ** *p* <.01. *** *p* <.001.

## Discussion

Consistent with the Activity Theory of Ageing [7], this study demonstrated a positive association between activity participation and well-being based on a sample of relatively active third-age individuals; thus, the findings may not be applicable to all aging adults. As documented in many studies [3,4], activity participation was related to greater subjective happiness. Meanwhile, the level of activity participation was found to predict better quality of life in third age adults, in agreement with the mainstream research findings [1,32–34].

This study supported the main postulation of the Social Production Function Theory [9–11] that financial status as an essential resource influences the relationship between activity participation and quality of life through a moderating effect, but did not confirm the direct impact of finance on subjective happiness and quality of life, which reflected the complexity of the relationship as asserted by the “Easterlin Paradox” [21,32]. It suggested that third age people with better financial status could benefit more in their quality of life from activity participation. In contrast, those with worse financial status might not have improved quality of life despite activity participation. A possible explanation could be that the major activities they participate in might be different from those with worse financial status [15]. It is undeniable that restful, enjoyable or meaningful activities are more helpful for their quality of life than household, unavoidable or involuntary ones [35]. In addition, as defined by the World Health Organization quality of life [32], as a subjective evaluation of the salience of each life sphere may vary in third age adults with different financial situations. Those with better financial status may value their activities more for their quality of life [36]. The difference could also be explained by the participants’ profiles. The results showed that participants with better financial status were younger, with higher education, and better physical and mental health. These strengths and related cognition capacity might make them more aware of the gains in activity participation.

The findings from this study suggest that the financial status of older populations should be considered in policy-making and service planning related to activity participation. It is essential to reduce the financial adversity of third age people through welfare policies, including pensions, insurance, allowances, and reliefs, among others. Inconsiderate activity interventions for third-age people may fail to achieve desired well-being outcomes. Effective measures should be taken to mitigate the impact of financial adversity on activity participation outcomes and to ensure improved quality of life and subjective well-being for all older activity participants. Firstly, pensions and insurance should be adjusted for inflation to enable older people to afford diverse activities. Secondly, in addition to basic living allowances, low-income older people could be provided with vouchers or kits for fee-based activities in communities or universities. These benefits could be distributed by community social workers during regular home visits to prevent stigmatization. Thirdly, older adults with poor or average financial status could receive transportation allowances or fee exemptions/reductions for activity participation through volunteer work. Fourthly, organized and meaningful activity programs should be tailored to the specific characteristics and capacities of third age adults with limited financial resources (e.g., upcycling, urban community planting, and city tours). Last but not least, tax exemptions could be granted to operators of activity facilities or programs to enhance affordability for third age people. These practices may largely reduce or eliminate the attenuating effect of financial adversity on the positive outcomes of activity participation. Moreover, public education campaigns on the benefits of activity participation could be conducted to enhance understanding among third-age people.

This study is limited in its cross-sectional nature and inability to reveal the causal relationships among variables. The relationships among activity, finance and well-being could be bi-directional, but this study only addressed the prediction of activity and finance on well-being. The sample source reduces its representativeness and generalizability, especially for homebound sedentary or solitary third age adults and those from regions with social and cultural backgrounds different from Hong Kong. Recall biases might have affected the retrospective reports of participants. Overall activity participation level was used to simplify the measurement, but it was unable to reflect the activity types participants with different financial status engaged in, which may account for their varied well-being outcome of activity participation. The measure of financial status was self-perceived, so it needs to be supplemented with objective measures (e.g., disposable income) in future studies. There could be important confounding variables omitted in the model, leading to specification errors.

## Conclusion

Active ageing has been recommended as a core coping approach for escalating population ageing. This study verified the applicability of established theories in the West (i.e., the Activity Theory of Ageing and the Social Production Function Theory) in the context of East Asia where social culture and welfare systems may be different. It adds to the knowledge about the positive impact of activity participation on the well-being of third age adults living in Asian societies, indicated by subjective happiness and quality of life specifically. At the same time, the financial status was found to moderate the relationship between activity participation and quality of life. It provides implications for all sectors working with third age people to attach importance to the influence of financial status and to take measures to eliminate the hindrances of financial adversity for third age populations to improve well-being via activity participation.
